# Health of the Newborn and Breastfeeding during the COVID-19 Pandemic: A Literature Review

**DOI:** 10.1055/s-0041-1741449

**Published:** 2022-01-31

**Authors:** Carmen Amelia Salvador-Pinos, Edson Zangiacomi Martinez, Susana Eulalia Dueñas-Matute, Rosa Romero de Aguinaga, Juan Carlos Jácome, Stephanie Michelena-Tupiza, Valheria Cárdenas-Morales

**Affiliations:** 1 Facultad de Ciencias Médicas, Universidad Central del Ecuador, Quito, Ecuador; 2 Department of Social Medicine, Ribeirão Preto Medical School, Universidade de São Paulo, Ribeirão Preto, SP, Brazil

**Keywords:** breast milk, COVID-19, microbiota, neonate, SARS-CoV-2, bacterial colonization, pregnancy, leite materno, COVID-19, microbiota, neonato, SARS-CoV-2, colonização bacteriana, gestação

## Abstract

**Objective** The present article presents a literature review concerning the microbiota of breast milk and the influence of epigenetics in the susceptibility to COVID-19.

**Methods** A literature review.

**Results** Breastfeeding transfers microbiota, nutrients, diverse white blood cells, prebiotics, hormones, and antibodies to the baby, which provide short- and long-term immunological protection against several infectious, gastrointestinal, and respiratory illnesses. The little evidence available shows that breast milk very rarely carries the SARS-CoV-2 virus, and even in those cases, it has been discarded as the source of contagion.

**Conclusion** The reviewed studies show evidence of a beneficial effect of breastfeeding and highlights its importance on the current pandemic due to the immune reinforcement that it provides. Breastfed individuals showed better clinical response due to the influence on the microbiota and to the nutritional and immune contribution provided by breast milk, compared with those who were not breastfed.

## Resumo

**Objetivo** O presente artigo apresenta uma revisão de literatura sobre a microbiota do leite materno e a influência da epigenética na suscetibilidade à COVID-19.

**Métodos** Revisão de literatura.

**Resultados** A amamentação transfere microbiota, nutrientes, diversos glóbulos brancos, prebióticos, hormônios e anticorpos para o bebê, os quais proporcionam proteção imunológica de curto e longo prazo contra diversas doenças infecciosas, gastrointestinais e respiratórias. As poucas evidências disponíveis mostram que o leite materno transporta muito raramente o vírus SARS-CoV-2, e mesmo nestes casos, ele foi descartado como fonte de contágio.

**Conclusão** Os estudos revisados mostram evidências de um efeito benéfico da amamentação e destacam sua importância na atual pandemia devido ao reforço imunológico que ela proporciona. Os indivíduos amamentados mostraram melhor resposta clínica devido à influência sobre a microbiota, e à contribuição nutricional e imunológica proporcionada pelo leite materno, em comparação com aqueles que não o foram.

## Introduction

There is increasing evidence supporting the benefits of breastfeeding, both for starting it as soon as possible and for maintaining it exclusively during the first stages of life. Even though most of the evidence consists of observational and comparative studies, supporting findings are steadily increasing in number and variety. Despite this, many concerns about the safety of breastfeeding have been raised due to the current COVID-19 pandemic. This has occurred even though the presence of the virus in breast milk has rarely been reported, and a route of infection has not been successfully demonstrated. 1 2 3 Therefore, the present article is a literature review concerning the microbiota of breast milk and the influence of epigenetics in the susceptibility to COVID-19.

## Methods

According to the typology presented by Grant et al., 4 the present article is a literature review including published materials that provide an examination of recent or current literature. The process for the present review adhered to the Search, Appraisal, Synthesis, and Analysis (SALSA) framework 4 : the completeness of searching was determined by scope constraints, there was no formal assessment of the quality of the studies, and a narrative and textual synthesis, as well as a thematic and descriptive analysis, were used.

## Results and Discussion

In a rapid systematic review including eight studies about pregnant women who were reverse transcription-polymerase chain reaction (RT-PCR)-positive for SARS-CoV-2 and presented with symptoms of the disease, no viral traits were found in breast milk nor in newborns. 5 Another systematic review evaluated the published evidence regarding the presence of SARS-CoV-2 in human milk. Only one study found some evidence of vertical transmission of the virus from mother to infant through breastfeeding, and only one other study attempted to find it directly in the milk, with negative results. 6 In addition, Dong et al. 7 found immunoglobulin G (IgG) and immunoglobulin A (IgA) in breast milk from an RT-PCR-positive mother, but other body fluids such as urine, vaginal secretion, and sweat were negative for the virus. Her baby was also negative when tested. Therefore, the authors suggested that breast milk plays a protective role against viral infection. 6 However, SARS-CoV-2-specific IgG levels in the newborn do not stay elevated for long periods (only for approximately a month a half), indicating a potential risk of being infected after this period. 6 The only reported cases of vertically transmitted infection from the mother to her newborn are related to intrauterine transmission. 8 9 In contrast, evidence suggests specific protection against SARS-CoV-2 inherited by the newborn through breast milk. 10 Therefore, breastfeeding should be maintained and highly recommended even during the pandemic, considering that the benefits outweigh the risks. 8 9 10

In addition to the extensively reported nutritional contribution of breast milk to newborns, a significant reduction in neonatal morbidity and mortality has been observed, especially in premature newborns. 8 9 10 11 This is most likely a result of the contribution of active and passive immunity. 12 Besides that, there is growing evidence on the influence that breast milk exerts on the formation of the microbiota of the neonate (intestinal, pulmonary, and other parts of the body), which extends the positive effects throughout the lifetime of the individual.

Detailed information on the active and passive immune contribution of breast milk has been continuously reported. Breast milk provides immune cells (leukocytes, neutrophils, eosinophils, monocytes, T and B lymphocytes, natural killer [NK] cells, immature granulocytes), IgA and IgG, free fatty acids, monoglycerides, proteins such as lactoferrin, oligosaccharides, and others. 12 All these components contribute to active immunity, stimulating the development of the immune system and of the intestinal mucosa. Immunoglobulin A and IgG in breast milk play a crucial role in direct and cross-protection against various pathogens. 13 Breastfeeding babies have shown a lower risk of developing asthma, inflammatory bowel disease, obesity, and diabetes, and that they have an improved humoral response to vaccination compared with formula-fed infants. The role of breast milk in the development of the infant microbiota is increasingly being acknowledged and has become relevant during the current pandemic. A prospective cohort study by CHILD-Canada collected information of 3,500 pregnant mothers and their children since 2009 and reported that breastfeeding positively influences the gut microbiota of the newborn. 14 Since this study, many others have published relevant information that has broadened our current understanding of this field and have suggested that healthy microbiota can be an additional tool against COVID-19. 15

Studies performed in Ecuador, as in many other developing countries, are primarily observational. For instance, Román Collazo et al. 16 suggest that breastfeeding for < 6 months may be associated with an increased risk of overweight and obesity in infants from Cuenca. Another study performed in Quito concluded that the levels of the immunoregulatory cytokine TNF in the breast milk of adolescent mothers were higher than those in adult mothers. 17 Thompson et al. 18 associated differences in the microbiota of the newborn with the birth route (cesarean section compared with vaginal delivery) in mothers from the Galapagos Islands. Reports suggesting a high probability of congenital infection of COVID-19 are scarce. One was reported in Toronto, Canada, 1 another in Tehran, Iran, 19 and one in San Luis Potosí, Mexico. 20 This last one reported negative results for SARS-CoV-2 in umbilical cord blood samples. Additionally, the virus or antibodies against it have been found in few samples of the placenta, amniotic fluid, and newborn blood. 11

The World Health Organization (WHO) states that 3 out of 46 mothers who had breast milk samples tested were positive for viral particles by RT-PCR but not for the live virus. 21 Among the three infants whose mothers were positive, only one was positive for SARS-CoV-2, but transmission through respiratory droplets or aerosols is the most plausible pathway of infection. In addition to the congenital infection reported in Toronto, 1 there is another in Ulm, Sweden, 14 that reported the complete virus in breast milk. The infant described in the Toronto study was born infected. 1 In the case from Ulm, the mother and her son had already been home for several days when both began to show symptoms of COVID-19, and the virus was found in the maternal milk for a few days. Thus, neither of the two cases showed a clear breast milk infection route. On the other hand, reactive IgA against SARS-CoV-2 in breast milk implies that breastfeeding promotes an additional defense against this disease. 11 However, it is unknown how the microbiota of the breast milk of COVID-19-positive mothers influences the neonates. This issue needs further investigation.

## Breastfeeding and the Immune System

During pregnancy, the embryo develops the first phases of the immune system, such as macrophages, T and B lymphocytes, neutrophils, interleukins, cytokines etc. This process is partially caused by the tolerance that both mother and embryo have. However, the system of the baby is poorly developed and has a weakened or nonexistent response compared with the adult immune system. For this reason, the neonate relies on the mother to increase its immune defense capacity after birth. Antibodies are actively transported from the mother to the embryo through the placenta, and the neonate is born with higher IgG than the mother, which serves as protection during the first months. 22 Similarly, during vaginal delivery, the neonate is exposed to 23 *Bacteroidetes* and *Actinobacteria* taxa that stimulate proinflammatory cytokines such as TNF and interleukin-18 (IL-18) by producing lipopolysaccharides. 23

After birth, breast milk is needed in order to continue this process. The colostrum provides chemokines, cytokines, immunoglobulins, growth hormone, and a variety of immune cells. 24 Because the lymphocyte populations found in breast milk are memory cells that have already faced pathogens, they will carry this information to the infant; therefore, directly providing protection. 25 26 In addition, the migration of maternal immune cells to the gastrointestinal mucous tissue of the neonate is suggested as another protective mechanism. The literature indicates that this process is more likely to occur during the early stages of lactation due to the high leukocyte concentrations found in this period, contributing to the development and to the correct maturation of the immune system in the intestinal mucosa. 26

Human milk oligosaccharides are indigestible by humans, but they work as prebiotics to stimulate the growth of bacterial species such as *Bifidobacterium* . These bacteria modulate the intestinal immune response and inhibit the adherence of pathogens to the intestinal epithelium, preventing pathogenic invasion and colonization from bacteria like *Campylobacter jejuni, Helicobacter pylori, Entamoeba histolytica* , and enterotoxigenic *Escherichia coli* . 27 Oligosaccharides may also contribute to an increase of short-chain fatty acids and essential molecules for signaling pathways in the gastrointestinal tract. 25 27 Additionally, breast milk contains extracellular vesicles that are high in mRNA and miRNA, which are essential for early metabolic regulation. This can enrich the metabolic processes of biosynthesis, glycolysis, gluconeogenesis, and fatty acid biosynthesis to enhance the functioning of the immune system and improve the establishment of healthy bacterial communities. 25

The exact reason for the low number of COVID-19 cases in children is not clear. One of the main theories involves the different expressions of the ACE 2 enzyme, which increases with age and is used by the virus to enter a cell. Another theory is the passive immunity from the mother, such as type III interferon, which has antiviral activity, demonstrated in a study on the Zika virus. 28 This immunity could act against SARS-CoV-2 without presenting the cytokinetic storm associated with COVID-19-positive patients with the worst clinical diagnosis.

## Microbiota and SARS-CoV-2

The relation between a healthy microbiota and a functional immune system is a theme that demands investigation in the current pandemic. A Chinese study reported that lung microbiota from COVID-19-infected patients showed alterations similar to those observed in patients with community-acquired pneumonia, 29 such as the predominance of pathogenic species and a reduction of *Lactobacillus* and *Bifidobacterium* . This is an indication of dysbiosis in the lung microbiota of COVID-19 patients. Gou et al. 30 studied the intestinal microbiota with proteomic biomarkers, finding an extremely close correlation with inflammatory cytokines that could suggest an individual predisposition to present with severe symptoms of COVID-19.

On the other hand, it has been reported that the administration of *Lactobacillus* isolated from breast milk to infants between 6 and 12 months old reduces the incidence of respiratory and gastrointestinal tract infections by between 30 and 40%. 31 32 Di Pierro 33 states that the species *Streptococcus salivarius* K12, a predominant commensal in the oral, pulmonary, and upper respiratory tract, has been safely used as a probiotic for 20 years now and has been correlated with a significant reduction in upper respiratory tract viral infections in children and adults.

SARS-CoV-2 RNA has been detected in fecal samples from children who recovered from COVID-19, 34 suggesting that the virus interacts with elements of the gut microbiota or, at least, with bacterial species of the digestive tract. A recent study including 15 acute/severe COVID-19 patients in Hong Kong through March 2020 found an increased abundance compared with controls of opportunistic intestinal pathogens known to cause bacteremia, such as *Clostridium hathewayi, Actinomyces viscous,* and *Bacteroides nordii,* regardless of gender, age, comorbidities, and use of antibiotics. 35 In contrast, beneficial bacteria, such as *Fecalibacterium prausnitzii, Lachnospiraceae bacterium, Eubacterium rectole, Ruminococcus obeum* , and *Dorea formicigenerans* , were greatly reduced in these cases. The researchers further concluded that there were microbiome profiles correlated with worse COVID-19 symptoms: bacteria of the genus *Coprobacillus* , known to increase ACE 2 expression in mice gut, *Clostridium ramosum* and *Clostridium hathewayi* . *Alistipes onderdonkii* , which is involved in the tryptophan-serotonin metabolism, is essential for gut homeostasis, and *Faecalibacterium prausnitzii* produces butyrate, being associated with a better prognosis. These findings support the need to assess the strengthening of microbiota as an additional tool for treating and preventing COVID-19. 36

## COVID-19 Epigenetics

In the current COVID-19 pandemic, the fact that a large percentage of infected people do not show any symptoms and that some show very mild symptoms, along with the wide variation of symptoms of those who manifest them, 37 has been a topic for conjecture. One plausible explanation can be found on epigenetics. Epigenetics is the study of heritable changes in the expression of the genome or phenotype, mainly caused by DNA methylation, phosphorylation, and acetylation to modify histones, noncoding RNA, ubiquitination, and other means. 38 The variety of organs affected by this virus in the human organism (lungs, brain, heart, kidneys, digestive system, vascular system, among others) raises many questions that could be answered by epigenetics. 39 40

In the past two and a half decades, one of the research topics in epigenetics was understanding the molecular mechanisms by which RNA viruses antagonize the signaling and detection components that regulate the induction of innate immune and antiviral defense programs in the host to an infection. Recently, increasing evidence supports the hypothesis that viruses, including lytic RNA viruses that replicate in the cytoplasm, have evolved to regulate the epigenome of the host cell and control the innate immune system in order to promote robust virus replication and pathogenesis. 41 Coronaviruses have been a field of research for > 15 years. A study on the 2003 outbreak of Severe Acute Respiratory Syndrome (SARS) caused by the SARS-CoV virus identified that the silencing of the *TICAM2* gene increases the susceptibility to SARS-CoV in mice. 42 This gene encodes a protein that helps activate Toll-like receptors (TLRs) involved in innate immunity, the first line of defense against pathogens.

## Methylation and COVID-19

For the virus to evolve, one of two pathways must be used: direct fusion with the host cell membrane or induction of host cells fusion. Both mechanisms facilitate viral endocytosis, invasion of neighboring cells, and evasion of the innate antiviral immune system. 43 The union of the virus with the cell forms a syncytium, a mechanism typical of coronaviruses and of many other viruses. 44 45 Syncytia formation normally occurs during the development of the mammalian placenta, in which syncytin genes are involved and produce syncytin-1 and -2 derived from two human endogenous retroviruses. 46 Additionally, syncytia formation leads to the creation of multinucleated giant cells in the placenta, making this tissue impermeable and generating mother-child immune tolerance. For this reason, syncytin genes, which are hypomethylated, are functionally active in the mammalian placenta. In other tissues, they are hypermethylated and, therefore, silenced. The activation of these genes in other tissues has been associated with various diseases, such as schizophrenia, multiple sclerosis, and type-1 diabetes. 47 48 For example, the Epstein-Barr virus and cytomegalovirus are known to demethylate the syncytin-1 and -2 genes of the host, increasing gene transcription and causing syncytia formation in tissues where those genes are normally hypermethylated and silenced. 29 31 Syncytia formation by SARS-CoV-2 is faster than that of SARS-CoV in 2002. Additionally, it is known that syncytia formation is highly responsible for the virulence factor and for the induction of a cytokine storm. 45 49

## Epigenetic Mechanisms in Susceptibility to SARS-CoV-2 and Severity of the Disease

Two recent studies 50 51 identified the importance of the gene encoding angiotensin-converting enzyme 2 (ACE2) methylation pattern and determined that its expression rate is under epigenetic control. 52 Corley et al. 50 showed that the activity of the *ACE2* gene, which is located on the X chromosome (Xp22.2), is associated with age and gender based on the methylation pattern of various CpG promoter islands. This gene is mainly present in the endothelium and the epithelium of many organs, such as the lungs and upper airways, the intestines, the liver, the kidneys, the heart, the eyes, and central nervous and vascular systems. 53 It was shown that the methylation rate in lung epithelial cells was the lowest compared with in all other tissues, suggesting that the lungs have the highest rate of *ACE2* transcription and expression. 53 It was also shown that age correlates with hypomethylation in lung tissue, which provides a partial explanation for aging being set as a significant risk factor for death by COVID-19. The opposite happens in children, in whom the *ACE2* gene in the lungs and other tissues is hypermethylated and, therefore, silenced. 54 This suggests that certain subgroups of patients with known epigenetic features are more susceptible to SARS-CoV-2, such as those with systemic lupus erythematosus (SLE), in whom a strong overexpression of the lung protein ACE2 seems to be related with hypomethylation. 55 Pique-Regi et al. 56 showed that the placenta expresses negligible amounts of both ACE2 and TMPRSS2, which could explain the scarcity of reported infections between mother and child in utero.

## Breast Milk as an Epigenetic Regulator

Breast milk has been considered an epigenetic regulator because of the ribonucleic acids derived from exosomes (miRNA), which can modulate the action of the DNA methyltransferase enzymes (DNMT). 57 These miRNAs are transferred by extracellular vesicles derived from the epithelial cells of the mammary gland and are inserted into somatic cells by endocytosis mechanisms. Breast milk also contains long-sequence noncoding RNAs known to be involved in regulating gene expression at the post-transcriptional level. Thus, its effect is not only restricted to DNA methylation. It is considered that consumption of human milk provides the appropriate epigenetic signals to the genetic programming of the newborn and is perhaps one of the mechanisms that protects against various respiratory conditions. 57 Regulatory exosomes of the immune function have been successfully isolated from breast milk. These miRNAs are known to increase the number of FoxP3 cells, also known as T cells (Tregs). Ribonucleic acid derived from exosomes binds to the enzymes DNMT1 and DNMT3b, which are associated with the FoxP3 locus on CD4 + T cells to mediate its expression. On the other hand, breast milk exosomes have also been associated with the function of the NR4A receptor family and with the expression of the nuclear factor NFKB, which can also regulate genes associated with the immune response by modulating DNMT. This combined evidence suggests that the microbiota of human milk may shape immune function through DNA methylation and other epigenetic changes. 57 58 During the COVID-19 pandemic, it would be promising to study the ability of miRNAs in human milk to epigenetically regulate the expression of various genes associated with the antiviral immune response, such as *HLA* , *IFN* , and, most importantly, the *ACE2* receptor.

## Epidrugs against SARS-CoV-2

Epigenetic regulation can be performed with natural interventions. An in-vitro study suggested using vitamin D and quercetin to improve the symptoms of COVID-19, 59 which would be done by inhibiting the expression of *ACE2* . Another study proposed that the average vitamin D level in European countries is inversely correlated with the COVID-19 death rate, but this study has several flaws in its statistical approach, some recognized by the authors themselves. 60 61 Thus, it should be carefully considered before openly recommending vitamin D to reduce the risk and symptoms of COVID-19. Another candidate for epigenetic silencing of the *ACE2* and interferon genes is curcumin. 62 Curcumin is a potent activator of DNA methyltransferases in viable clinical doses and has ferritin-reducing effects. 63 64 65 This becomes important when considering that high ferritin values in patients with COVID-19 are associated with greater severity of the disease. 66 Another so-called epidrug with proven methylation capability is sulforaphane from broccoli. 67 All of these substances are natural, over-the-counter medicines and may help reduce the severity and susceptibility of the disease. Pathways such as HIF-1 signaling, autophagy, RIG-I signaling, toll receptor signaling, fatty acid oxidation/degradation, Il-17 signaling, and others have been found to be sequestered or suppressed by viral proteins leading to improved viral survival. 68 Furthermore, pathways such as relaxing signaling in the lungs suggest that aberration by viral proteins could lead to the pulmonary pathophysiology found in COVID-19. The epigenetic profile of the host tissue could allow the discovery of risk factors for COVID-19 related to age and gender.

## Genetic Susceptibility

Genetic susceptibility refers to the increased likelihood of developing a particular disease based on an individual's genetic makeup. Genetic population studies show important variations in protein-coding between different ethnic groups, making some of them more susceptible to diseases. This might also help understand why most children are unaffected by COVID-19, while a few still develop serious symptoms. 69 The *ACE2* gene encodes a protein of 805 amino acids that exhibits observable variabilities between different ethnic groups, 70 71 72 an interesting topic in this pandemic. There are three genetic patterns related to predisposition to COVID-19. 73 The first one involves common variants at multiple loci that have a weak effect with a unique contribution. However, they can show a significant effect through additive contribution, increasing the risk and severity of infection. Second, moderately rare variants in limited genes usually have strong effects and, therefore, would be highly penetrating and even dominant. These variants could be the basis for severe forms in young patients without any other medical conditions. Lastly, gene-environment interactions could define the predisposition of the host. The genetic variants related to the immune system that involve the previous strain of coronavirus may have a role in the susceptibility to infection, since SARS-CoV-2 has a genetic identity of 80% with the previous virus. Previous studies have supported the role of human leukocyte antigen (HLA) in the susceptibility and severity of various viral infections. 74 Therefore, the genetic variability of the three main genes of the histocompatibility complex (MHC) class I (HLA A, B and C) could also be involved in the susceptibility and severity of SARS-CoV-2. Similarly, a synonymous variant in the IFN-induced transmembrane protein 3 gene has been reported to cause severe clinical outcomes in patients infected with the H7N9 and H1N1 influenza viruses. Likewise, it should be noted that the *Mannose-Binding Lectin* , *CD147* , *CCL2* , and *interleukin-12* genes should also be considered regarding the susceptibility to COVID-19. 75

## Recommendations Regarding Breastfeeding and COVID-19

It should be emphasized that, until now, we know with certainty that there is no vertical transmission from a SARS-CoV-2 infected mother to her child and that breastmilk acts as a protective element against this viral agent. 76 The WHO, The International Federation of Gynecology and Obstetrics, and The American and Spanish Academies of Pediatrics highlight the considerable benefits of breastfeeding both for the mother and the child, even during critical conditions like the actual COVID-19 pandemic. 77 In June 2020, the protocol “Recommendations for health professionals from Human Milk banks regarding the attention of pregnant women, mothers in puerperium and breastfeeding mothers, premature or low-weight newborns with or without risk of infection of SARS-CoV-2 COVID-19” was published in Ecuador. In this document, breastfeeding and the support that milk banks can provide to mothers are promoted for those who might not feel safe breastfeeding or might not be in optimal conditions to do so. The permanent use of personal protective equipment, handwashing for at least 20 seconds, wearing a face mask, and keeping a two-meter safe distance between individuals are essential measures to avoid the spread of the disease to and from mother, child, and the health professionals taking care of them. 78

## Conclusion

The reviewed studies show evidence of a beneficial effect of breastfeeding and highlight its importance on the current pandemic due to the immune reinforcement that it provides. Breastfed individuals showed better clinical response due to the influence of breastfeeding on microbiota and to its nutritional and immune contribution compared with those who were not breastfed. Even though a few studies found SARS-CoV-2 fragments and one publication found the full virus in breast milk, a route of infection through it has not been established. This information needs to be updated as new evidence is published. On the other hand, the relation between a healthy microbiota, especially derived from breast milk, and a positive response to COVID-19 might suggest novel therapeutic options by strengthening the microbiota. The genetic susceptibility of certain individuals could be modified by little known epigenetic factors in which the microbiota could play a key role through the interaction with the genome of the individual and with SARS-CoV-2. Further experimental research needs to be performed in order to improve the clinical guidelines in SARS-CoV-2-positive patients.
